# Rapid deployment of SARS-CoV-2 testing: The CLIAHUB

**DOI:** 10.1371/journal.ppat.1008966

**Published:** 2020-10-28

**Authors:** Emily D. Crawford, Irene Acosta, Vida Ahyong, Erika C. Anderson, Shaun Arevalo, Daniel Asarnow, Shannon Axelrod, Patrick Ayscue, Camillia S. Azimi, Caleigh M. Azumaya, Stefanie Bachl, Iris Bachmutsky, Aparna Bhaduri, Jeremy Bancroft Brown, Joshua Batson, Astrid Behnert, Ryan M. Boileau, Saumya R. Bollam, Alain R. Bonny, David Booth, Michael Jerico B. Borja, David Brown, Bryan Buie, Cassandra E. Burnett, Lauren E. Byrnes, Katelyn A. Cabral, Joana P. Cabrera, Saharai Caldera, Gabriela Canales, Gloria R. Castañeda, Agnes Protacio Chan, Christopher R. Chang, Arthur Charles-Orszag, Carly Cheung, Unseng Chio, Eric D. Chow, Y. Rose Citron, Allison Cohen, Lillian B. Cohn, Charles Chiu, Mitchel A. Cole, Daniel N. Conrad, Angela Constantino, Andrew Cote, Tre’Jon Crayton-Hall, Spyros Darmanis, Angela M. Detweiler, Rebekah L. Dial, Shen Dong, Elias M. Duarte, David Dynerman, Rebecca Egger, Alison Fanton, Stacey M. Frumm, Becky Xu Hua Fu, Valentina E. Garcia, Julie Garcia, Christina Gladkova, Miriam Goldman, Rafael Gomez-Sjoberg, M. Grace Gordon, James C. R. Grove, Shweta Gupta, Alexis Haddjeri-Hopkins, Pierce Hadley, John Haliburton, Samantha L. Hao, George Hartoularos, Nadia Herrera, Melissa Hilberg, Kit Ying E. Ho, Nicholas Hoppe, Shayan Hosseinzadeh, Conor J. Howard, Jeffrey A. Hussmann, Elizabeth Hwang, Danielle Ingebrigtsen, Julia R. Jackson, Ziad M. Jowhar, Danielle Kain, James Y. S. Kim, Amy Kistler, Oriana Kreutzfeld, Jessie Kulsuptrakul, Andrew F. Kung, Charles Langelier, Matthew T. Laurie, Lena Lee, Kun Leng, Kristoffer E. Leon, Manuel D. Leonetti, Sophia R. Levan, Sam Li, Aileen W. Li, Jamin Liu, Heidi S. Lubin, Amy Lyden, Jennifer Mann, Sabrina Mann, Gorica Margulis, Diana M. Marquez, Bryan P. Marsh, Calla Martyn, Elizabeth E. McCarthy, Aaron McGeever, Alexander F. Merriman, Lauren K. Meyer, Steve Miller, Megan K. Moore, Cody T. Mowery, Tanzila Mukhtar, Lusajo L. Mwakibete, Noelle Narez, Norma F. Neff, Lindsay A. Osso, Diter Oviedo, Suping Peng, Maira Phelps, Kiet Phong, Peter Picard, Lindsey M. Pieper, Neha Pincha, Angela Oliveira Pisco, Angela Pogson, Sergei Pourmal, Robert R. Puccinelli, Andreas S. Puschnik, Elze Rackaityte, Preethi Raghavan, Madhura Raghavan, James Reese, Joseph M. Replogle, Hanna Retallack, Helen Reyes, Donald Rose, Marci F. Rosenberg, Estella Sanchez-Guerrero, Sydney M. Sattler, Laura Savy, Stephanie K. See, Kristin K. Sellers, Paula Hayakawa Serpa, Maureen Sheehy, Jonathan Sheu, Sukrit Silas, Jessica A. Streithorst, Jack Strickland, Doug Stryke, Sara Sunshine, Peter Suslow, Renaldo Sutanto, Serena Tamura, Michelle Tan, Jiongyi Tan, Alice Tang, Cristina M. Tato, Jack C. Taylor, Iliana Tenvooren, Erin M. Thompson, Edward C. Thornborrow, Eric Tse, Tony Tung, Marc L. Turner, Victoria S. Turner, Rigney E. Turnham, Mary J. Turocy, Trisha V. Vaidyanathan, Ilia D. Vainchtein, Manu Vanaerschot, Sara E. Vazquez, Anica M. Wandler, Anne Wapniarski, James T. Webber, Zara Y. Weinberg, Alexandra Westbrook, Allison W. Wong, Emily Wong, Gajus Worthington, Fang Xie, Albert Xu, Terrina Yamamoto, Ying Yang, Fauna Yarza, Yefim Zaltsman, Tina Zheng, Joseph L. DeRisi

**Affiliations:** 1 Chan Zuckerberg Biohub, San Francisco, California, United States of America; 2 University of California San Francisco, Department of Microbiology and Immunology, San Francisco, California, United States of America; 3 University of California San Francisco, School of Medicine, San Francisco, California, United States of America; 4 University of California San Francisco, Department of Biochemistry and Biophysics, San Francisco, California, United States of America; 5 University of California San Francisco, Institute for Neurodegenerative Diseases, San Francisco, California, United States of America; 6 University of California San Francisco, Division of Infectious Disease, San Francisco, California, United States of America; 7 Howard Hughes Medical Institute, Chevy Chase, Maryland, United States of America; 8 University of California, Berkeley, California, United States of America; 9 University of California San Francisco, Department of Experimental Medicine, San Francisco, California, United States of America; 10 University of California San Francisco, Department of Laboratory Medicine, San Francisco, California, United States of America; 11 Gladstone Institute, San Francisco, California, United States of America; 12 eSix Development, Oakland, California, United States of America; 13 Joint Bioengineering Graduate Program, University of California, Berkeley, California, United States of America; University of Pittsburgh, UNITED STATES

Accurate diagnostic testing is at the center of controlling infectious disease outbreaks. It allows clinicians and public health practitioners to identify and treat infected individuals as well as inform larger public health interventions (such as quarantine) to prevent new infections. The emergence of the Severe Acute Respiratory Syndrome Coronavirus 2 (SARS-CoV-2)/Coronavirus Disease 2019 (COVID-19) pandemic in the human population in November 2019 has spotlighted the importance of diagnostic testing as a (foundational) bedrock to understand how infections occur and spread. Remarkably, multiple studies now show that asymptomatic and presymptomatic individuals are drivers of community transmission [1]. Thus, broad testing capability is necessary to quell resurgence of outbreaks and reopen the global economy.

Laboratory barriers to testing include the limited availability of space, high-capital infrastructure (robotics and information systems), material resources with uninterrupted supply chains, and trained personnel qualified to conduct assays in Clinical Laboratory Improvement Amendment (CLIA)-approved laboratories. However, sizable untapped capacity exists throughout the United States in a multitude of university research laboratories. Theoretically, this capacity could be directed to rapidly expand emergency response capabilities; however, real barriers exist to their entry into clinical diagnostic testing. Research laboratories—while working with the same suite of methods and from the same biological principles as clinical diagnostic laboratories—have substantively different workflows, expectations of quality control and validation, information management, documentation, traceability, reporting, and regulatory compliance. For research scientists, this burden may appear extremely formidable.

To address the dearth of testing availability in the San Francisco Bay Area, the Chan Zuckerberg Biohub, in partnership with the University of California, San Francisco (UCSF) Clinical Microbiology Lab, rapidly built and deployed an emergency COVID-19 viral testing facility, staffed by a combination of Biohub employees and UCSF graduate students, postdocs, and faculty. These efforts were facilitated by guidance from the Food and Drug Administration (FDA) on February 29, 2020, “Policy for Coronavirus Disease-2019 Tests During the Public Health Emergency” [2], and an Executive Order (March 12, 2020) from California State Governor Gavin Newsom modifying certain California-specific regulatory personnel requirements [3]. The goal of this facility, called the “CLIAHUB,” was to provide free, rapid, high-capacity testing services to local hospitals, Department of Public Health offices, and community-based clinical screening efforts in the San Francisco Bay Area, with a particular emphasis on underserved populations. In this perspective piece, and in the accompanying document [4], we describe in detail the experiences of the Biohub, including our organizational structure, recruitment and training of highly skilled volunteers, automation, information management, engineering, protocol development, and clinical validation. We share the lessons we learned and offer guidance to other research laboratories pursuing rapid expansion of diagnostic capabilities in the context of emergency response for COVID-19 and future pandemic responses.

The Chan Zuckerberg Biohub is a nonprofit research institute that employs approximately 110 people organized into multiple smaller research groups, with nearly half of the staff focused on infectious disease research. The organization’s strong focus on collaborative work, communication, and open science allowed frictionless redistribution of personnel priorities into COVID-19 work. On March 12, 2020, the Biohub’s COVID-19 response staff was organized into 13 work teams (staff members from various research groups and seniority levels were appointed as Team Leads and Backup Team Leads according to their skills and experience). To ensure continuity in the event of an outbreak, laboratory infection control procedures were developed, and all job functions and critical knowledge were held by at least 2 individuals. Communications were facilitated by daily all-hands meetings and dedicated Slack channels. Once clinical testing was underway, volunteers from UCSF were trained to run the assay and organized into 4 nonoverlapping squads to reduce viral transmission risk.

Clinical laboratories are required to meet all applicable federal, state, and local regulations in order to perform patient testing and have to be certified with their state and the Center for Medicare and Medicaid Services (CMS) under CLIA. These regulations specify the laboratory organizational structure and minimum operational standards, with major areas covering the laboratory environment, staffing roles and requirements, information tracking and management, testing validation and procedures, quality assurance, and proficiency testing. Each of these areas had to be addressed with compliance documentation as the CLIAHUB was formed. Ensuring regulatory compliance required close collaboration between the CLIA laboratory director, microbiology section director, and clinical quality assurance and Biohub laboratory lead staff. Since the CLIA laboratory director is ultimately responsible for the operations and quality of test results reported by the laboratory, the importance of this close partnership between the UCSF clinical laboratory and the Biohub cannot be overstated.

**Fig 1:**
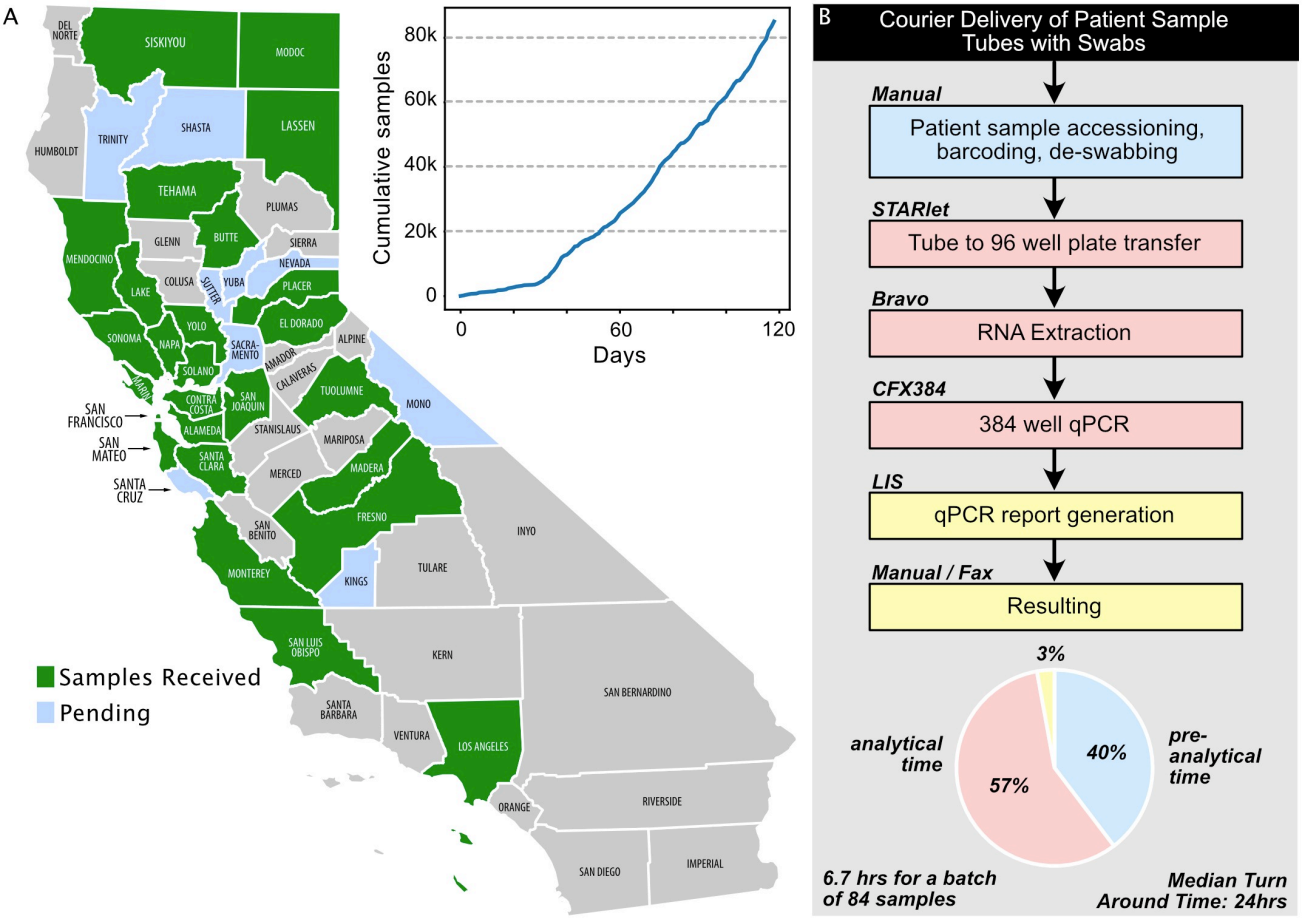
(A) Counties served by CLIAHUB and the growth of testing volume over the first 120 days of operation. The most frequent users of testing at CLIAHUB were the surrounding Bay Area counties; however, outlying areas, such as Placer and Lassen, were also frequent users despite the distance. (B) CLIAHUB testing workflow and approximate timing are shown on normalized to a single sample, for preanalytical (blue), analytical (red), and postanalytical (green/yellow). The time to process a batch of 84 samples was approximately 6.7 h, including preanalytical and postanalytical steps. Median TAT was 24 h, highlighting the variability in extrinsic factors not under laboratory control, such as medical courier delivery times. qPCR, quantitative polymerase chain reaction; TAT, turnaround time.

The complete CLIAHUB workflow is detailed in Fig 1B and the linked document. Briefly, the CLIAHUB workflow begins with medical courier or shipping company delivered patient samples (nasopharyngeal (NP) or oropharyngeal (OP) swabs). In order to preserve RNA integrity and maximize sensitivity, DNA/RNA Shield was chosen for the transport media. After receipt, patient information was recorded (“accessioned”), and tubes were barcoded, de-swabbed, and queued for transfer into 96 well plates. Transfer from tubes to plates was achieved using both do-it-yourself (“DYI”) engineered and commercial automated solutions. Eight stations using high-speed liquid handlers were used to extract viral RNA, after which reverse transcription polymerase chain reaction (RT-PCR) was run using probes for both the N and E gene of SARS-CoV-2. All reagents and samples were tracked using a custom serverless laboratory information system (LIS). After assay optimization, limit of detection (LOD) was established by serial dilution of a quantified high-titer patient sample, and validation of the assay for the Emergency Use Authorization (EUA) application was achieved by retesting samples previously identified as positive or negative based on a previously approved assay at the UCSF clinical lab.

**Fig 2:**
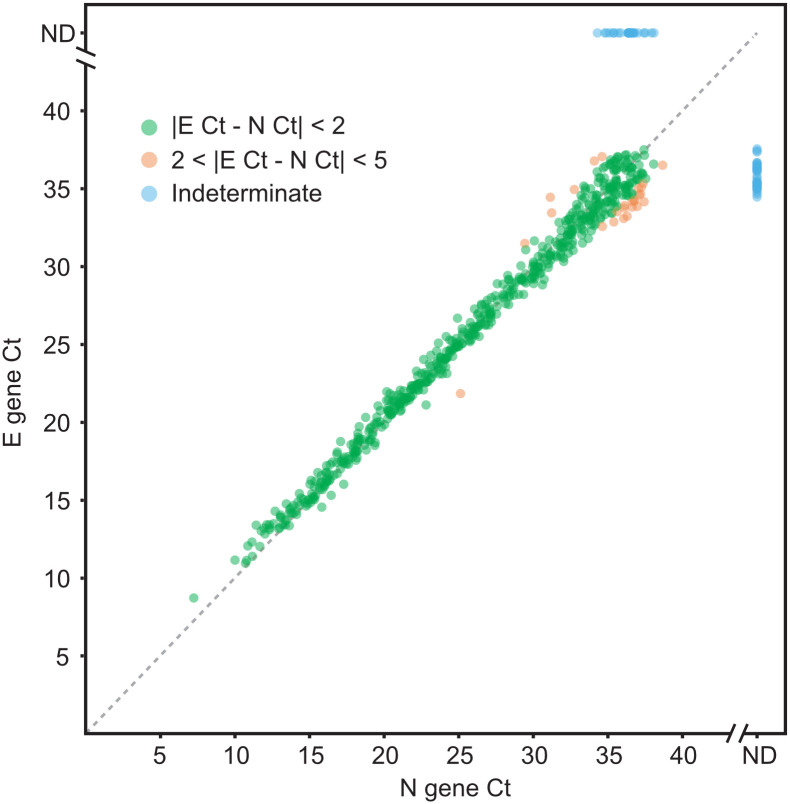
Concordance between RT-PCR Ct values for the E and N gene probes. For 91% of samples, agreement between viral gene probe sets were within 5 Ct cycles, with the majority within 2 cycles. For 9% of samples, only 1 gene was detected (5% E gene only, 4% N gene only), leading to an indeterminate result. In all such cases, the sample extraction and RT-PCR was repeated; points plotted represent the retest result. Ct, cycle threshold; ND not detected; RT-PCR, reverse transcription polymerase chain reaction.

On March 20, 2020, 8 days after starting this project, the CLIAHUB returned its first clinical result. As of this writing (October 17, 2020), the CLIAHUB has returned **151,795** clinical results. A total of 36 California county Department of Public Health (“DPH”), safety net hospitals, jails, skilled nursing facilities, and public clinics have utilized the CLIAHUB for SARS-CoV-2 testing (Fig 1), in addition to community-based studies. In the period from May 8 to June 8, 2020, the CLIAHUB processed over 1,300 samples on average per day. The maximum number of samples processed in a single day was 2,688. Median turnaround time (TAT) was 24 h. For genomic epidemiology, positive samples were routed for full SARS-CoV-2 genome sequencing [1]. While the samples processed by CLIAHUB were heterogeneous and not representative of the general population, several important characteristics of the disease and the test were notable. A broad range of cycle threshold (Ct) values have been observed (from 7 to 45), indicative of a tremendous range of viral loads within this disease. Samples were considered positive if the Ct for both the N and E gene probes were less than 40 for the original standard operating procedure (SOP). If a sample was positive for 1 of the 2 probes, the result was considered indeterminant. In subsequent iterations of the protocol, samples were considered positive if either of the 2 probes were below a Ct of 40. While there was an excellent agreement between both viral probes, in 9% of cases, only 1 of the 2 probes were detected, suggesting that assays that rely on detection of a single gene alone may have a significant false negative rate (Fig 2).

The largest users of CLIAHUB testing capacity have been Bay Area and outlying county DPHs, including San Mateo, Marin, Alameda, Placer, Tehama, Santa Clara, and Sonoma counties. Feedback from individual counties indicated that access to free and fast testing allowed insight into at-risk populations, which, in turn, led to deployment of medical personnel. In the case of congregate care facilities, intervention and stabilization avoided evacuation of residents to local hospitals. The CLIAHUB also directly enabled community-based studies in the town of Bolinas, the Mission District, and Bayview Hunters Point neighborhoods of San Francisco, and a study of unsheltered persons [1]. These studies provided both clinically actionable results for individuals and communities, in addition to valuable insight about the socioeconomics and demographics of populations that were disproportionately affected. Altogether, the experience of CLIAHUB demonstrates that research facilities can indeed be converted to clinical testing laboratories during a pandemic emergency declaration that are capable of returning high-quality and timely clinical results in order to meet the challenge of a growing pandemic. We hope that the accompanying “How to” document, protocols, and engineering details (dx.doi.org/10.17504/protocols.io.bfi2jkge) will serve as a guide for others in the midst of this or any other future pandemic.
